# Anacardiaceae Family: Effect of Isolated Compounds and Other Identified Phytochemicals Against Clinically Relevant *Candida* Species—A Short Review

**DOI:** 10.3390/antibiotics14030308

**Published:** 2025-03-17

**Authors:** Rosane Nassar Meireles Guerra, Aluísio Silva Oliveira, Josivan Regis Farias, Danielle Cristine Gomes Franco, Pamela Gomes Santos, Nicolle Teixeira Barbosa, Simone Batista Muniz, Afonso Gomes Abreu, Flavia Raquel Fernandes Nascimento

**Affiliations:** Laboratorio de Imunofisiologia, Programa de Pós-Graduação em Ciências da Saúde, Universidade Federal do Maranhão, Campus Bacanga, Av. dos Portugueses, 1966, São Luís 65080-805, Brazil

**Keywords:** antifungal, alpha-pinene, *Candida albicans*, estragole, flavonoids, gallic acid

## Abstract

**Background:** The increased rates of common fungal diseases are a constant challenge. Therefore, the search for plant-based compounds with antifungal activity, particularly ones against *Candida* species, is always relevant in the medical context. However, most of the studies have focused on screening the antifungal activity of extracts rather than isolated compounds. Based on this, we aimed to analyze and organize a comprehensive overview of the antifungal and other biological activities of isolated compounds found in Anacardiaceae family vegetal species, covering mechanisms of action and potential therapeutic applications. **Results:** The extracts, essential oils, and compounds are frequently assayed for anti-*Candida* activity using the in vitro minimum inhibitory concentration (MIC), minimum fungicide concentration (MFC), and halo inhibition assays. *Candida albicans*, *C. tropicalis*, *C. parapsilosis*, *C. glabrata*, *C. krusei*, and *C. guilliermondii* were the most tested fungus species. Essential oils were the most used form (37% of the studies). The isolated compounds included shikimic acid, 2-hydroxy-1,8-cineole β-D-glucopyranoside, myricitrin, cardanol, estragole, trans-anethole, β-caryophyllene, myrcene, catechin-3-O-rhamnoside, *β*-sitosterol-3-O-glucoside, 24Z-isomasticadienolic acid, oleanolic acid, pistagremic acid, apigenin, sakuranetin, oleanolic aldehyde, and integriside. **Conclusions**: Our data indicate that the compounds isolated from Anacardiaceae species show promise for developing new therapeutic antifungal drugs, mainly if we consider their other biological activities, including anti-inflammatory, antioxidant, and apoptotic effects. In this context, they may be candidates for future treatments of fungal infections, especially in combination with conventional antifungals or when used in nanostructured formulations, which may result in a new avenue of using plant extracts and isolated compounds.

## 1. Introduction

The epidemiology of invasive candidiasis varies geographically, with a significant increase in diversity worldwide. It accounts for the highest rate of mortality and hospital-acquired infections, mainly among newborns, the aging, and immunosuppressed patients [1]. According to estimates, *Candida* spp. is the most frequent genus, and candidiasis causes 400,000 new cases annually in the United States. In the same way, mortality rates related to *Candida* sp. infections remain high in Brazil [2] and worldwide despite therapeutic advances, including the introduction of echinocandins [3]. This high index of *Candida* infections increases the period of hospitalization, economic burden, and mortality, especially in ICU patients, those undergoing chemotherapy, or patients submitted to complex therapeutic interventions and abdominal surgeries. Another critical question is the increased resistance to available antifungal and several mechanisms of fungal virulence and resistance [3,4].

*Candida albicans* is the primary cause of candidiasis and the most prevalent species identified in severe infections, even after increased diseases caused by related species classified as non-albicans [3,5]. *Candida albicans* is part of the commensal microbiota and can colonize various human tissues as a commensal organism, working as a barrier in synergism with the innate immune system [6]. However, host-related factors and an imbalance in the microbiota can break homeostasis, affecting tissue integrity or leading to host immune response defects. Altogether, these alterations can predispose the transformation of harmless *Candida* into an opportunistic pathogen, causing superficial candidiasis, which can progress into invasive mycoses of deep organs and systemic infections. Once established, these systemic infections become a severe public health problem [5] since they are responsible for long periods of hospitalization and high levels of mortality.

Recently, the species collectively classified as non-albicans have been identified in several nosocomial infections. They include *C. glabrata*, *C. parapsilosis*, *C. tropicalis*, and *C. krusei* [5,6]. All *Candida* species showed invasive capacity and are associated with systemic infections, especially *C. glabrata*, *C. parapsilosis*, and *C. auris.*

The invasive capacity of *Candida* sp. is influenced by several virulence factors, including its ability to switch from yeast to hyphal forms, adhesion, and biofilm formation. Adhesion is an important virulence factor because it contributes to host tissue invasion, favors hyphae growth, and increases the production of extracellular polymers that provide a structural matrix and facilitate biofilm formation in biological substrates or synthetic materials [6,7,8,9].

The molecular and cellular mechanisms behind the fungal resistance may involve several virulence factors, including increased efflux that reduces the drug accumulation and the biodisponibility within the cell, mutations in target protein genes, and changes in metabolic pathways, such as alterations in ergosterol biosynthesis, which is crucial for the structure and function of the fungal cell wall. Moreover, biofilm formation can further enhance resistance, increasing morbidity rates [10]. The emergence of antifungal failure in clinical medicine can also be related to an altered drug uptake, drug target alternation, and/or the fungus mechanism to drug inactivation [8,9]. The expression of one or several escape mechanisms favors resistance to commercial antifungals, impairing the treatment of candidiasis.

Currently, therapeutic options for *Candida* infections are limited to five main classes of compounds: polyenes, allylamines, azoles, fluoropyrimidines, and echinocandins [11]. However, despite their frequent application, some commercial drugs are limited because of their high toxicity at high doses, fungal tolerance, and fungal resistance, which underscores the necessity for novel therapeutic agents [12,13]. For this reason, the design and development of renewable resources frequently receive remarkable attention and efforts to identify new therapeutic strategies and discover new candidates from synthetic compounds and natural products as antifungal drugs [11,14,15] using in vitro, in vivo, and in silico tests. Indeed, computational simulation can be a helpful step in drug rational design and screening [16,17].

The focus on isolated compounds from the Anacardiaceae family is noteworthy. Anacardiaceae species have broad distribution, accessibility, and applications of their products. This family encompasses economically and medicinally significant species such as mango (*Mangifera indica*), cashew (*Anacardium occidentale*), and pistachio (*Pistacia vera*), which are found in diverse climatic regions, ensuring their wide availability [18]. Moreover, many species within the Anacardiaceae family contain bioactive compounds with antimicrobial, antifungal, immunological, and antioxidant properties, making them highly valuable in the pharmaceutical, cosmetic and agricultural industries. Their cultivation is also relatively cost-effective, as many of these species are already widely farmed for food production globally.

The limited pipeline of new antifungal drugs further exacerbates the emergence of multidrug-resistant fungal pathogens for several *Candida* species, including *Candida auris* [11], prompting the need for a comprehensive review of emerging treatment options and novel drug targets based on isolated compounds from the Anacardiaceae family. Studying the anti-*Candida* activity of isolated compounds from the Anacardiaceae family offers significant value from both scientific and therapeutic perspectives. This includes the ethnopharmacological use of these plants and the diverse range of bioactive compounds, many of which have known antifungal properties, that have been previously documented in the literature.

Based on this, this review aims to provide a concise yet thorough overview of the antifungal and other related biological activities of isolated compounds from Anacardiaceae species, focusing on their mechanisms of action and potential therapeutic applications, particularly for combating *Candida* infections. In addition, this review offers a unique opportunity to compile and highlight several of these bioactive compounds in one place, potentially contributing to the discovery of effective and safe antifungal agents, considering that the bioactive potential of Anacardiaceae species could play a significant role in bioprospecting efforts in the search for new antifungal solutions and the development of sustainable, affordable treatments.

## 2. The Anacardiaceae Family

The Anacardiaceae family includes approximately 81 genera and 800 species worldwide, often in tropical and subtropical regions. It is economically significant because it provides edible fruits (mango, cashew, pistachio, and others), wood, and ornamental plants [18].

The family comprises woody plants with resiniferous ducts and glabrous or hairy branches. The leaves are simple, composite, or pinnate, without stipules and arranged alternately. The flowers, frequently arranged in terminal inflorescences, are pentamerous, actinomorphic, and have a super ovary, four to five sepals, a syncarpous gynaeceum, and free stamens. They may be unisexual, bisexual, or polygamous, and the color varies among the different species, including white, greenish, or purplish. The fruits are usually of the drupe type. In Brazil, 55 species are distributed in 14 genera, and the most diverse are *Schinus* (11 species) and *Anacardium* (9 species) [19].

Some species of the Anacardiaceae family are used in traditional medicine due to their antifungal activity, including *Rhus typhina* L. [20], *Anacardium occidentale* L. [21,22,23], *Cottinus coggyria* Scop. [24,25] *Lannea kestingii* Engl. and K. Krause [26,27], *Mangifera indica* L. [28], *Pistacia* spp. [29,30,31,32,33,34,35,36,37,38,39], *Rhus* spp. [20,40,41], *Schinopsis brasiliensis* Engl. [42], *Schinus* spp. [43,44,45,46,47,48], and *Spondias* spp. [49,50,51].

Species of the Anacardiaceae family are of great importance due to their use as food (genera *Anacardium, Mangifera*, and *Pistacia*) or due to several biological activities found in other uses. In addition, the Anacardiaceae family, owing to its unique chemical compositions, includes bioactive metabolites such as terpenoids, flavonoids, bioflavonoids, and alkyl and alkenyl phenols [14]. This indicates that the family species are an important source of novel bioactive compounds with therapeutic properties and industrial applications [18].

## 3. Anacardiaceae Species with Anti-*Candida* Activity

After analysis of the articles, we identified 35 studies reporting anti-*Candida* activity of vegetal species from the Anacardiaceae family. Table 1 summarizes the plant species listed in alphabetical order and their respective extracts/fractions and plant parts used. The table also shows the chemically characterized and isolated compounds, the *Candida* species or strain used in the studies, the type of assay, and methods used to assess anti-Candida activity. The Latin species names were validated at World Flora Online (WFO) [52]. It is vital to clarify that each species’ identity by the plant taxonomist(s) is reported only in Table 1.

This family has medicinal plants used in traditional medicine to treat infections in several countries [53,54,55]. The results identified 21 species belonging to 9 genera of Anacardiaceae with anti-*Candida* activity. The most prevalent genus was *Pistacia*, with eleven studies [29,30,31,32,33,34,35,36,37,38], and *Schinus*, with seven studies [43,44,45,46,47,48]. However, in terms of species, *Anacardium occidentale* [21,22,23], *Pistacia atlantica* [29,30,31], and *Pistacia lentiscus* [34,35,36] were the most investigated, with three studies each, followed by *Cotinus coggyria* [24,25], *Lannea Kerstingii* [26,27], *Rhus typhina* [20,41], and *Spondias tuberosa* [50,51], with two studies each. Other species also showed anti-*Candida* effects, including *Mangifera indica* [28], *Rhus coriaria* [40], *Schinopsis brasiliensis* [42], and *Spondias mombin* [49].

Essential oils were the most frequently used form, reported in 37.1% (*n* = 12) of the studies. Extracts and essential oils were obtained from leaves (54%), bark (17%), seeds (9%), fruits (9%), hulls (9%), flowers (6%), roots (3%), and nutshells (3%).

*C. albicans* was the most frequently tested fungal species, accounting for 74% of the studies. It was followed by *C. tropicalis* (23%), *C. parapsilosis* (17%), *C. glabrata* (11%), *C. krusei* (11%), and *C. guilliermondii* (4%).

The anti-*Candida* activity of extracts and compounds was frequently determined by halo inhibition on microbiological media (21%), minimum inhibitory concentration (MIC −89%), and minimum fungicidal concentration (MFC −21%). Only two studies evaluated the effect on biofilm formation, with one study on exoenzymes (proteinase and phospholipase) and one study on the growth curve. It is essential to highlight that only one study conducted in vivo tests in rats to evaluate the anti-*Candida* activity in a model of vulvovaginal candidiasis, and the remaining studies performed in vitro assays.

The MIC-based criteria to classify the antifungal activity are not uniform, and the MIC values have several interpretations, with a high range of values from 12.5 μg/mL [27] to 60 mg/mL [29]. For Barbosa et al. [54], values equal to or below 500 μg/mL were potent inhibitors of fungal activity. However, for other authors [55], compounds with MIC ≤ 1000 μg/mL displayed weak antifungal activity, whereas MIC values between 10 and 100 μg/mL denoted high antifungal activity [47,56]. For this reason, the MIC values must be used as initial screening instead of a definitive result to confirm the anti-*Candida* activity of one determined product In addition, most studies on the susceptibility of *Candida* spp. have followed the Clinical and Laboratory Standards Institute (CLSI) [57] to determine MIC and MFC using broth dilution. However, the standard that establishes these methods was developed to test antimicrobials with already-known parameters, leading us to conclude that there is an urgent need to create new procedures to evaluate plant extracts with antifungal and antimicrobial activity.

Other antifungal methods included halo inhibition, growth curves, and morphological transition [50]. Only a few studies assessed the antifungal activity against virulence factors such as adhesion, biofilm formation, or the production of exoenzymes, and no studies used investigated the effect of isolated compounds using in vivo experimental models.

Identifying bioactive compounds in plant extracts for experimental purposes comprises a series of essential steps, including determining the quality and quantity of the compounds considering the choice of solvent, extraction method, phytochemical screening procedure, fractionation method, and identification technique [15,16,57]. Our results showed that chemical analysis frequently involved the extraction of essential oils and the investigation of their antifungal activity [24,25,27,28,29,31,38,40,42,45,48]. In a study by Donati et al. [42], these compounds were isolated from the essential oil of *Schinopsis brasiliensis* and showed fungicidal activity against *C. parapsilosis*.

According to Donadu et al. [58], *Ruta graveolens* essential oil, which contains 2-undecanone as its main component, showed antifungal activity against fluconazole-resistant *C. tropicalis* and partially removed *C. albicans* biofilms. The time-kill kinetics assay revealed a fungicidal effect against *C. tropicalis* and a fungistatic activity against *C. albicans*. The authors also found a synergistic effect for the essential oil when combined with amphotericin B. These findings reinforce that natural products and isolated substances could be used as adjuvants to commercial antifungals to improve anti-*Candida* treatments.

The effects of anti-*C. albicans* and anti-*C glabrata* were tested against pistagremic acid, apigenin, and sakuranetin, the isolated compounds from *Pistacia chinensis* subsp. *integerrima*. Apigenin was more effective against *C. albicans* than the extract, showing a percent zone of inhibition of 29.32% for miconazole, but it was ineffective against *C. glabrata* [32].

## 4. Mechanisms Associated with the Anti-*Candida* Activity of Some Isolated Compounds Found in the Anacardiaceae Family

**Figure 1:**
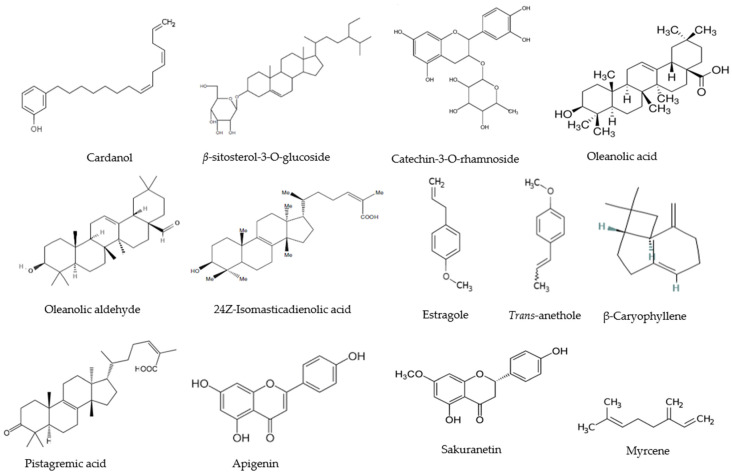
Chemical structure of some identified and isolated compounds in extracts from plants of the family Anacardiaceae with antifungal activity against *Candida* spp.

The anti-*Candida* activity of the isolated compounds has been assessed in vitro. Among them the following can be cited: cardanol [23], *β*-sitosterol-3-O-glucoside [26], gallic acid, benzoic acid [25], *β*-sitosterol-3-O-rhamnoside [27], catechin-3-O-rhamnoside [27], nilocitin, 1,3-di-O-galloyl-β-D-4, C1-glucopyranose [30], pistagremic acid, apigenin, sakuranentin [32], integriside A, integriside B [33], 24Z-isomasticadienolic acid, oleanolic acid and oleanolic aldehyde [34], α-pinene, α-terpineol, camphene, D-limonene, and 3-carene [38], as well as estragole, myrcene, trans-anethole, and β-caryophyllene [42] (Table 2). Figure 1 shows the chemical structure of the isolated compounds.

The high prevalence of *Candida albicans* in the studies reviewed may be attributed to its frequent detection in infections, particularly vulvovaginitis [59], and in critically ill COVID-19 patients admitted to intensive care units [60]. Another critical factor is accurately identifying other *Candida* species [60]. While phenotypic methods are commonly used for identifying yeasts in clinical samples, significant disparities exist between low-income and wealthier countries regarding diagnostic resources. As a result, microscopic examination of fungal structures is still widely employed despite its limited sensitivity. More advanced techniques, such as MALDI-TOF mass spectrometry and real-time PCR-based methods, offer greater accuracy in rapid fungal identification but are also more expensive [61].

It is essential to highlight that the pathogenicity of *C. albicans* and other *Candida* species is driven by multiple virulence factors and immune evasion mechanisms. These include adhesion, biofilm formation, the secretion of hydrolytic exoenzymes, and increasing resistance or tolerance to antifungal treatments [8,9]. Collectively, these characteristics present a significant challenge for healthcare providers in managing *Candida* infections. However, in our analysis, no studies specifically evaluated the effects of isolated compounds on these virulence factors.

Among the plant species investigated for their anti-*Candida* activity, *Anacardium occidentale* [21,22,23], *Pistacia lentiscus* [35,36,37], and *Schinus polygamus* [43,45,46] were the most frequently studied, mainly through extracts obtained from aerial parts. These extracts demonstrated efficacy against various *Candida* species. However, despite promising in vitro findings, studies evaluating the in vivo anti-*Candida* effects of these extracts and their identified compounds or isolated active substances remain scarce in the literature.

Our study identified a broad range of chemically characterized compounds within these plant species, even though their antifungal activity was not directly assessed in many cases. The most frequently reported substances in the *Anacardiaceae* family were gallic acid [20,21,22,25,30,39,41,43,50], α-pinene [24,25,31,36,38], and limonene [24,38,46,48]. According to D’Arrigo [38], a combination of isolated compounds—such as α-pinene, α-terpineol, camphene, D-limonene, and 3-carene—extracted from *Pistacia vera* exhibited more vigorous anti-*Candida* activity than any of these compounds individually.

Below, we discuss some key isolated compounds and their reported biological activities.

### 4.1. Cardanol

Cardanol is an isoprenoid phenolic acid isolated from cashew nutshell and *Rhus thyrsiflora* Balf.f, which can act against *Candida* sp. through different biological pathways and cellular targets than existing antifungal agents [62]. The antifungal activity of cardanol was related to its ability to bind to chitin on the yeast cell wall [23,29]. In addition, cardanol is considered one of the most promising byproducts of the industry and technological innovations [63], including the production of antimicrobial and antifungal paints [64]. However, despite the biological activity, this compound shows a potent cytotoxicity [65], which limits its application due to a lack of biocompatibility and makes its use as a therapeutic agent in treating candidiasis unfeasible.

### 4.2. Oleanonic Acid Oleanonic Aldehyde and 24Z-Isomaticadionolic Acid

Species belonging to the genus *Pistacia* are essential to many communities’ nutrition and agricultural economy. This genus has been extensively studied in botany, ethnobotany, phytochemistry, and pharmacological activity [66,67]. However, only the studies conducted by Rauf et al. [32], Irfan et al. [33], and Karygianni et al. [34] showed anti-*Candida* activity for isolated compounds from *Pistacia* spp. Other authors have identified the anti-*Candida* activity of those compounds by studying other vegetal species [65,66].

Mastic gum is a product found in several species of the *Pistacia* genus. It contains a high-molecular-weight polymer with healing and antimicrobial activities, *cis*-1,4-poly-β-myrcene [67]. Mastic gum exhibits an anti-plaque effect on dental surfaces, inhibits *Helicobacter pylori*, and shows antimicrobial action on several bacteria species [68].

Oleanonic acid and oleanonic aldehyde show antifungal and anti-inflammatory activities related to the activation of 5-lipoxygenase during in vivo studies. The anti-inflammatory and antioxidant effects of oleanonic aldehyde are related to its modulatory activity under the peroxisome proliferator-activated receptor (PPAR) on macrophage activation and cytokine production [66,69], as those effects are crucial to candidiasis control [34,69,70]. In this case, the antifungal effect of this compound may be potentiated by its immunomodulatory activity, since the immune response is essential to controlling the infection.

Oleanonic acid is a pentacyclic triterpene with a keto group at C-3 responsible for increasing this biological activity. This compound can inhibit biofilm formation and may exert this antifungal activity through several mechanisms acting alone or in combination. The main effects include inhibiting ergosterol, which disrupts cell membrane integrity, causes cell leakage, and causes fungal death. In addition, this compound may inhibit glucan and chitin synthase, making the fungal cell more susceptible to osmotic stress by increasing reactive oxygen species (ROS). This can lead to oxidative damage, affecting the metabolic process critical for fungal survival [71].

24Z-isomaticadionolic acid, a natural tetracyclic triterpenoid, showed an anti-inflammatory effect in chronic inflammations mediated by leukotriene B4. This compound also showed inhibitory activity against Gram-positive and Gram-negative bacteria [34].

### 4.3. α-Pinene

The antifungal activity of *Pistacia lentiscus* against *Candida albicans* and *Candida glabrata* has been attributed to the presence of α-pinene and terpinene-4-ol, both of which were identified in the essential oil extracted from its leaves [35]. Similarly, the hulls of *Pistacia vera* [38], and the essential oil derived from the leaves of *Schinus terebinthifolius* [47] exhibited antifungal activity against *C. albicans*, which is also linked to the presence of α-pinene. 

α-Pinene, a hydrocarbon monoterpene, is primarily found in essential oils and the hulls of *Pistacia* species. This compound possesses notable anti-inflammatory and antioxidant properties [72]. Its anti-inflammatory effects are associated with its ability to modulate key inflammatory cytokines, including tumor necrosis factor-α (TNF-α) and interleukin-6 (IL-6) [73]. Regarding antifungal activity, α-pinene has demonstrated efficacy against *C. albicans*, *C. parapsilosis*, and *C. glabrata* [38]. Additionally, it has been effective against clinical isolates of *C. albicans* and *C. parapsilosis* obtained from otomycosis, both when used alone and in combination with boric acid [74] or conventional antifungal agents [75]. The mechanism underlying its antifungal effects is likely related to its ability to inhibit the morphological transition from yeast to pseudo-hyphae and its capacity to reduce ballistoconidia formation. These effects enhance its potential for controlling Candida infections [64].

### 4.4. Gallic Acid

Gallic acid is a phenolic compound widely known for its antimicrobial and immunological potential. Gallic acid salts and esters, called gallates, are widely distributed in plants and found in A. occidentale bark [21,23]. This compound can reduce the morphological transition of *C. albicans* to filamentous forms, modify the mitochondrial transmembrane potential, increase the production of reactive oxygen species, and modify the membrane permeability, resulting in the apoptosis of fungal cells [76]. Evidence shows that liposomes containing quercetin and gallic acid can inhibit fungus growth. In addition, gallic acid’s anti-*C. albicans* activity improved survival in a murine model of systemic infection and showed antioxidant and anti-inflammatory properties [77]. Other studies confirm gallic acid’s promising role as an antifungal agent for treating multidrug-resistant *Candida* species, especially when combined with azoles [78]. Gallic acid is effective as an antifungal against planktonic and biofilm cultures of *C. albicans*, *C. glabrata*, and *C. tropicalis* when used in concentrations of clinical relevance. This is achieved through the interference of ergosterol biosynthesis, a crucial step in fungal cell membrane formation [43,62,78]. An in vivo study showed that treatment with gallic acid improved the survival of mice lethally infected with *C. albicans* with activity comparable to that of fluconazole [79].

### 4.5. β-Sitosterol-3-O-Glucoside and Catechin-3-O-Rhamnoside

*β*-Sitosterol-3-O-glucoside and catechin-3-O-rhamnoside, compounds isolated from the stem bark of *Lannea kerstingii*, exhibited activity against *C. albicans* and *C. tropicalis* [26]. *β*-sitosterol-3-O-glucoside showed antiapoptotic activity [80], and catechin-3-O-rhamnoside has antioxidant [81], anti-inflammatory, and anticancer properties [82] in plant species belonging to other families. Our in silico investigation showed that *β*-sitosterol-3-O-glucoside has high drug activity potential as an antifungal, as the activity value was 0.722 and the inactivity was 0.009 using SwissADME (http://www.swissadme.ch, accessed on 25 October 2024).

Catechin-3-O-rhamnoside is a flavonoid glycoside found in various plant species, including *Lannea kerstingii* [27]. This compound showed antimicrobial properties against multiple pathogens, including methicillin-resistant *Staphylococcus aureus*, *Escherichia coli*, *Klebsiella pneumoniae*, and *Candida* species. The anti-*Candida* activity exhibited inhibition zones ranging from 22.0 to 35.0 mm, with minimum inhibitory concentrations (MICs) of between 6.25 and 12.5 µg/mL. In addition, this flavonoid showed antioxidant properties [26]. These findings suggest that catechin-3-O-rhamnoside possesses significant antimicrobial and antioxidant properties, highlighting its potential for therapeutic applications. According to Stanislaus et al. [27], the antifungal activity may be due to its ability to complex with bacterial and fungal extracellular and soluble proteins.

### 4.6. Estragole and Trans-Anethole

Estragole isolated from essential oils extracted from the leaves of *S. brasiliensis* is a phenylpropanoid compound found in various essential oils and exerted activity against *C. parapsilosis* [41]. One proposed mechanism for the antifungal effect is related to the capacity to induce oxidative stress within fungal cells. Synergistic activity between estragole and ketoconazole has been reported against *C. tropicalis*. The time-kill curves showed significant synergism between the medicine and the isolated compound in this case. In contrast, the combination with amphotericin B had an antagonistic effect and was ineffective, and the fungus remained alive [83]. A study investigating the impact of estragole, along with related compounds eugenol and methyl eugenol, on *Candida albicans* revealed that these compounds elevated levels of reactive oxygen species (ROS) and compromised the antioxidant defense system of the fungi, leading to cell death [84]. Additionally, essential oils containing estragole have demonstrated antifungal activity against various fungal pathogens. For example, the essential oil of *Foeniculum vulgare* (fennel), rich in estragole, has shown effectiveness against fungal strains, suggesting a potential role of estragole in disrupting fungal cell membranes or interfering with ergosterol synthesis [85].

A checkerboard study reported the anti-*Candida* effect of trans-anethole by Dąbrowska et al. [86]. Trans-anethole antifungal properties are not fully elucidated. However, the authors proposed that trans-anethole may interact with fungal cell membranes, leading to increased permeability and loss of essential cellular components, mainly when combined with conventional antifungal drugs.

### 4.7. Myrcene

Myrcene is one of the main compounds found in essential oils of plant species such as *Cotinus coggyria*. It exhibits activity against *C. albicans* and *C. parapsilosis* [24]. However, according to our in silico results, this compound shows high toxicity. Given its tumorigenic, irritant, and harmful reproductive effects (-), this may explain why no data are related to its biological activity. Interestingly, anacardic acid, considered a marker of the Anacardium genus, was not included as an antifungal agent or identified in the studies evaluated in this review. However, Anacardic acid has other biological activities, such as antibacterial [21], immunomodulatory, and anti-inflammatory [87].

### 4.8. Apigenin

The antifungal activity of this natural flavone is related to the ability to induce morphological changes in *Candida albicans*, especially cell shrinkage, by altering the fungal membrane potential. Apigenin also induces membrane dysfunction, increasing cell permeability. This activity is essential to increasing the antifungal activity since it may result in leakage of intracellular components. Apigenin antifungal activity was also related to its interference with mitochondrial calcium signaling [87]. In addition, apigenin altered the fungal growth kinetics, reduced the adhesive properties, inhibited enzymes, and induced morphological changes. According to Lee et al. [88] apigenin changed the cytosolic calcium levels. This activity can favor the cleavage of members of the BCL-2 family, which can lead to cell damage caused by apoptosis.

### 4.9. Terpinen-4-ol

Terpinen-4-ol exhibited potent activity against both azole-susceptible and -resistant *Candida albicans* strains. The minimum inhibitory concentration (MIC) values were approximately 0.06% *v*/*v*, indicating its efficacy irrespective of the strains’ resistance profiles [89]. In a rat model of vaginal candidiasis, terpinen-4-ol reduced fungal infection caused by azole-resistant *C. albicans* strains. The antifungal efficacy of terpinen-4-ol is attributed to its ability to disrupt fungal cell membranes [90].

### 4.10. Pistagremic Acid and Sakuranetin

There are few data related to the anti-Candida activity of pistagremic acid. Our search shows that only Rauf et al. [32] described the anti-*Candida* activity of pistagremic acid. The pistagremic acid isolated from the galls of *Pistacia integerrima* is a triterpene with many biological properties determined by in vitro and in vivo studies. The activity as an antifungal was determined in vitro against *C. albicans* and *C. glabrata.* The range of halo inhibition assay was 29 to 32 mm for *C. albicans* and 15 to 42 mm for *C. glabrata*, varying according to the strain of each species.

Sakuranetin inhibits the activity of efflux pumps in *Candida* sp., which can increase the efficacy of antifungal agents. In addition, this compound disrupts the synthesis of β-glucan, weakens the fungal cell membrane, and increases the susceptibility to osmotic stress and cell lysis. Sakuranetin is an anti-inflammatory flavonoid, an activity that can contribute to its antifungal effect.

### 4.11. Integrisides A and B

These two compounds are new acylated oligosaccharides from *Pistacia integerrima*. Their antifungal effect is probably related to the electrostatic interactions across the positively charged chitosan (oligosaccharides) and the negatively charged cell surface, leading to destabilization of the cell membrane, which leads to the leakage of cells [33].

## 5. Delivery Systems as an Alternative to Reduce Toxicity and Improve Antifungal Action

The delivery system is an interesting strategy to reduce the toxicity of isolated compounds and simultaneously enhance their effectiveness as an antifungal agent. The conjugation of vegetal compounds with biocompatible molecules, such as polyethylene glycol (PEG), chitosan, or PLGA (poly(D, L-lactide-co-glycolide), can improve its solubility in aqueous environments and facilitate safer and more efficient delivery. In addition, both delivery systems can reduce toxicity and improve the pharmacokinetic properties of bioactive compounds (Kolge et al., 2023 [91]). Nanotechnology-based drug delivery systems, particularly those utilizing nanoparticles, present an innovative and adaptable approach in the pharmaceutical field, since these delivery systems can more effectively target fungal cells and act on biofilms since they can enter the fungal cells. Additionally, these systems can be engineered to release the drugs or isolated compounds in a controlled manner, reducing the risk of high local concentrations and toxicity.

Polyethylene glycol, a polymer of ethylene oxide monomers, is safe and non-toxic. It has been approved by the FDA for human use [92] and has been used in several areas in clinical trials [93]. PEGylation involves modifying the therapeutics by linking one or more PEG + molecules to increase the pharmacokinetic and pharmacodynamic properties. For example, PEGylation prolongs the half-life of gentamicin by 7–15 fold [94], enhances the solubility of curcumin [95], and significantly increases the water solubility of sybilin [96].

Polymeric PLGA nanoparticles have garnered significant attention as drug carriers due to their ability to enhance bioavailability, solubility, and therapeutic efficacy while minimizing toxicity and enabling sustained drug release. Composed of lactic and glycolic acid monomers, PLGA undergoes rapid biodegradation within the body. It has been widely explored for improving the oral bioavailability of drugs and mitigating the nephrotoxicity associated with amphotericin B, a potent antifungal agent [97]. The multifunctionality of PLGA nanoparticles was further demonstrated when the encapsulated voriconazole exhibited greater antifungal potency against *Candida albicans* compared to its free form [98].

Another noteworthy polymer in drug delivery is chitosan, a naturally derived, linear cationic polysaccharide consisting of D-glucosamine and N-acetyl D-glucosamine units [99]. Chitosan has emerged as a valuable excipient due to its mucoadhesive properties, ability to enhance permeation, and capability of controlled drug release. Additionally, it is recognized for its biodegradability, biocompatibility, and safety profile. Its cationic nature facilitates interactions with anionic polymers, including alginate, carbopol, and PLGA, further broadening its applications in drug delivery systems [100].

## 6. Challenges and Future Directions

The antifungal potential of Anacardiaceae extracts is promising, but challenges remain in optimizing their efficacy and safety for clinical use. One option includes the development of delivery systems. However, although chemical modification and the possibility to produce delivery systems using liposomes, microspheres, and nanoparticles can enhance stability and decrease the clearance of drugs and diagnostics, they are not readily applicable to proteins, peptides, or other biologicals [92]. For this reason, the main target for future research may focus on elucidating the molecular mechanisms of action, optimizing formulations, and conducting pre-clinical and clinical trials to evaluate and determine the therapeutic potential of these plant-based antifungal agents. Meanwhile, only a few studies have assessed the efficacy of bioactive compounds as anti-Candida agents or the viability of extraction at the industrial level.

At the laboratory level, enough studies have been published identifying and isolating the compounds in the Anacardiaceae species and their potential applications in the pharmaceutical and cosmetic industries. Still, another significant challenge is the broadcast of growth for the sectors related to the demand for bioactive compounds, and the development of advanced drugs for therapeutic industrial use is positive. These two steps are essential to allow their use in clinical trials.

Anacardiaceae plants, including *Schinus terebinthifolius*, *Anacardium occidentale*, *Spondias purpurea*, and *Pistacia* species, among others, exhibit significant anti-*Candida* sp. properties. For this reason, these plants offer promising avenues for developing new antifungal treatments, with some enhanced effects when combined with other agents or delivery systems. However, challenges remain in optimizing the efficacy and safety of those active principles and producing them on a large scale for clinical use. An interesting perspective is the development of delivery systems, such as the nanostructured lipid system used in experimental tests, considering the possibility of enhancing the bioavailability, reducing toxicity, and increasing the effectiveness of vegetal extracts and isolated compounds. Another possibility is to use the extracts with conventional antifungals as adjuvant therapy. These strategies can enable those substances to be developed as a more viable antifungal agent, acting more effectively against the virulence factors.

## 7. Conclusions

In conclusion, this review’s findings indicate that future research should focus on further elucidating the molecular mechanisms of action, optimizing formulations, and conducting in vivo tests and preclinical and clinical trials to establish the therapeutic potential of these plant-based anti-*Candida* sp. as antifungal agents. These steps are the future avenue in plant-based antifungal drugs as sustainable therapeutic options for preventing or mitigating fungal infections and associated morbidity and mortality.

## Figures and Tables

**Table 1:** Extracts and compounds of plants of the family Anacardiaceae with anti-*Candida* activity (2012 to 2024).

Plant Species	Type of Extract or Fraction (Plant Part)	Compounds Identified and/or Isolated/Reference	*Candida* Species Tested	Type of Assay (Methods) *	Ref.
*Anacardium occidentale* L.	Ethanolic (flowers, leaves, stem bark)	Phosphoric acid, dodecanoic acid, ethylgallic acid, sorbitol, glucose, gallic acid, hexadecanoic acid, octadecanoic acid, 1,2-benzenedicarboxylic acid	*C. albicans* *C. tropicalis*	In vitro (halo inhibition, MIC, MFC)	[21]
	Ethanolic (bark)	Galloyl-beta-glucose, gallic acid, epicatechin gallate, luteolin, agathisflavone, 7: 9,12,13-trihydroxyoctadec-10-enoic acid, caffeoyl-d-glucose	*C. albicans*, *C. krusei*, *C. tropicalis*	In vitro (MIC/MFC), growth curve	[22]
	(NI) * Cashew nutshell	Cardanol **	*C. albicans*	In vitro (MIC)	[23]
*Cotinus coggyria* Scop	Essential oil (leaves)	α-pinene, β-pinene, limonene, α-terpinolene, β-terpinene, β-myrcene, β-caryophyllene, limonene β-phellandrene, β-ocimene, t-terpinene, o-cymene	*C. albicans* *C. parapsilosis*	In vitro (halo inhibition)	[24]
	Ethyl alcohol (leaves and flowers)	Gallic acid **, benzoic acid **, rutin, ferulic acid, quercetin, hyperoside, disulphuretin, sulphuretin, kaempferol, sulphurein, 7-O-β-D glucopyranoside, apigenin, pentagalloyl glucose, methyl gallate, 3-*O*-α-L-rhamnofuranoside	*C. albicans*	In vitro (halo inhibition)	[25]
*Lannea kerstingii* Engl. and K. Krause.	Ethyl acetate (stem bark)	*β*-sitosterol-3-O-glucoside **	*C. albicans*, *C. krusei*, *C. tropicalis*	In vitro (halo inhibition, MIC, MFC)	[26]
		Catechin-3-O-rhamnoside **	*C. albicans* *C. tropicalis*	In vitro (MIC, MFC)	[27]
*Mangifera indica* L.	NI (peel and seed)	Proanthocyanidins, gallates, gallotannins	*C. bracarensis*, *C. glabrata* *C. parapsilosis*, *C. nivariensis*	In vitro (halo inhibition MIC)	[28]
*Pistacia atlantica* Desf.	Essential oil (leaves, fruits)	α-Pyrene, terpinen-4-ol acid	*C. albicans*	In vitro (MIC)	[29]
	Methanolic (leaves)	Nilocitin **, 1,3-di-O-galloyl-β-D-4 **, C1-glucopyranose **, gallic acid, ellagic acid, gallotannins, 3,3’-dimethoxyellagic acid, 2,3-di-O-galloyl-(α/β)-4 C1-glucopyranose, 1,2,3,4,6-penta-O-galloyl-β-D-4	*C. albicans*	In vitro (halo inhibition)	[30]
*Pistacia atlantica* subsp. *kurdica*	Essential oil (hulls)	α-Pinene, β-citral, carvone hydrate, myristic acid, *p*-acetyltoluene, pinocarveol, palustrol + 88 compounds	*C. albicans*	In vitro (halo inhibition, MIC)	[31]
*Pistacia chinensis* subsp. *integerrima*	Fresh galls	Pistagremic acid **, apigenin **, sakuranetin **	*C. albicans*, *C. glabrata*	In vitro (MIC)	[32]
*Pistacia integerrima*	Aerial parts	Integriside A **; integriside B **	*C. albicans*, *C. glabrata*	In vitro (halo inhibition, MIC)	[33]
*Pistacia lentiscus* L.	Mastic gum	24Z-isomasticadienolic acid **, oleanolic acid **, oleanonic aldehyde**	*C. albicans*	In vitro (MIC)	[34]
	Oils (seeds)	α-Pinene terpinen-4-ol, limonene, β-myrcene, caryophyllene linoleic acid, oleic acid, fatty acid, β-sitosterol, protocatechuic acid, *p*-coumaric, t-cinnamic + other compounds	*C. albicans*	In vitro (halo inhibition)	[35]
	Essential oil (leaves)	α-Pinene, terpinen-4-ol, camphene D-limonene 3-carene, and 60 other compounds	*C. albicans* *C. glabrata*	In vitro (MIC)	[36]
	Polyphenol enriched MeOH extract (leaves)	Shikimic acid, 2-hydroxy-1,8-cineole β-D-glucopyranoside, myricitrin **	*C. albicans*	In vitro (growth rate)	[37]
*Pistacia vera* L.	Essential oil (hulls)	α-Pinene **, α-terpineol **, camphene **, D-limonene **, 3-carene **	*C. albicans*, *C. parapsilosis* *C. glabrata*	In vitro (MIC, MFC, checkboard, time-kill curve)	[38]
		Gallic acid, cyanidin-3-O-galactoside, catechin, epicatechin, eriodictyol-7-O-glucoside, naringin, eriodictyol, quercetin, naringenin, luteolin, kaempferol	*C. albicans* *C. glabrata* *C. parapsilosis* *C. auris*	In vitro (MIC)	[39]
*Rhus coriaria* L.	Essential oil (seeds)	Linoleic acid, oleic acid, palmitic acid	*C. albicans*	In vitro (halo inhibition, MIC)	[40]
*Rhus typhina* L.	Hydroalcoholic extract, essential oil (branches, leaves, and fruits)	Gallic acid, 1-cyclohexane-3,4,5- hydroxy-carboxylic acid, malic acid, d-cadinene, β-pinene, phenylacetaldehyde	*C. albicans*	In vitro (halo inhibition, MIC)	[20]
	Ethanolic (leaves and berries)	Gallic acid, chlorogenic acid, gentisic acid, sinapic acid, caffeic acid, ethyl gallate	*C. albicans*	In vitro (MIC)	[41]
*Schinopsis brasiliensis* Engl.	Essential oil (leaves)	Estragole **, *trans*-anethole **, β-caryophyllene ** myrcene	*C. parapsilosis*	In vitro (MIC)	[42]
*Schinus lentiscifolius* Marchand.	Aqueous, *n*-hexane, ethyl acetate, and *n*-butanol fractions (leaves)	Nonadecanol, moronic acid, gallic acid, quercetin, quercitrin	*C. albicans*, *C. tropicalis*	In vitro (MIC)	[43]
*Schinus molle* L.	Volatile oil dried leaves	Spathulenol, β-caryophyllene, caryophyllene oxide	*C. albicans*, *C. glabrata*, *C. krusei*, *C. orthopilosis*, *C. parapsilosis*, *C. rugosa*, *C. tropicalis*, *C. metapsilosis*	In vitro (MIC)	[44]
	Petroleum ether, diethyl ether, acetone, aqueous (leaves)	Sesquiterpenes, sesquiterpenoids, and other terpenes	*C. albicans*	In vitro (halo inhibition; MIC)	[45]
*Schinus polygamus* Cav	Essential oil (bark)	*dl*-limonene, myrtenal, caryophyllene oxide	*C. albicans*	In vitro (MIC)	[46]
	Essential oil (leaves)	E-caryophyllene, DL-limonene β-pinene	*C. albicans*	In vitro (MIC)	[46]
	Essential oil (leaves and fruits)	A-phellandrene, β-phellandrene, α-pinene, germacrene D	*C. albicans*, *C. tropicalis*, *C. krusei*, *C. guilliermondii*, *C. parapsilosis*	In vitro (MIC)	[43]
*Schinus terenbintifolius* Raddi	Essential oils (leaves and fruits)	Monoterpene hydrocarbons, α-pinene camphene, β-pinene terpinolene, β-phellandrene	*C. albicans*	In vitro (MIC)	[47]
*Schinus weinmannifolius* Engl.	Essential oil (leaves)	Bicyclogermacrene, limonene	*C. albicans*	In vitro (MIC)	[48]
*Spondias mombin* L.	Aqueous (leaves), hydroethanolic (bark)	Quercetin, caffeic acid, catechin, kaempferol	*C. albicans* *C. tropicalis*	In vitro (MIC, MFC)	[49]
*Spondias tuberosa* Arruda.	Hexane (leaves)	Gallic acid, fatty acids	*C. albicans*, *C. parapsilosis*, *C. glabrata*, *C. krusei*	In vitro (MIC, MFC)	[50]
	Hydroalcoholic (leaves and roots)	Dehydroascorbic acid, quinic acid, and others	*C. albicans*, *C. tropicalis*	In vitro (MIC, morphological transition)	[51]

(*) NI was not informed in the article; MIC—minimum inhibitory concentration; MFC—minimum fungicidal concentration. (**) Isolated compounds tested for anti-*Candida* activity.

**Table 2:** Isolated compounds found in Anacardiaceae species with anti-Candida activity.

Plant Species	Plant Part	Chemical Method	Compounds Isolated	Com. Conc. ^a^ μg/mL	Drug ^b^ (μg/mL)	MIC ^c^ (μg/mL)	Disk/Hallo (mm)	Ref.
*Anacardium* *occidentale*	Cashew nutshell	High performance liquid chromatography (HPLC), NMR, MALDI-TOF, and others	Cardanol	512–1.0	NI ^d^	64	-	[23]
*Cotinus coggyria*	Leaves and flowers	Chromatographic column, preparative HPLC	Gallic acid, benzoic acid	NI	FZL	-	13 ± 0.5	[25]
*Lannea kerstingii*	Stem bark	Liquid and thin-layer chromatography	*β*-sitosterol-3-O-glucoside,	200	FZL 50	50	-	[26]
		Thin-layer chromatography 1H NMR	catechin-3-o-rhamnoside	50 to 6.25 µg/mL	FZL 50	12.5	22–35	[27]
*Pistacia chinensis*	Galls	Mass spectroscopy	Pistagremic acid, apigenin, sakuranetin	NI	MCZL AmphoB	-	19 ± 1.0% 29 ± 0.4% 36−42 ± 0.7%	[32]
*Pistacia integerrima*	Aerial parts	Liquid chromatography reverse-phase CC preparative HPLC	Integriside A, integriside B	NI	MCZL	93–95 89–92	30 30	[33]
*Pistacia lentiscus*.	Mastic gum	Liquid chromatography (MPLC)	24Z-isomasticadienolic acid, oleanolic acid, oleanonic aldehyde	2.4–2500	NI	1250 1250 1250	- - -	[34]
*Pistacia vera*	Hulls	GC-FID and GC-MS analysis	α-Pinene, α-terpineol, camphene, D-limonene, 3-carene	NI	VOZL (0.0156–16) FZL (0.0625–64) CFGN (0.00195–2)	>1000 >1000 >1000 125−250 62.5–250	- - - - -	[38]
*Schinopsis brasiliensis*	Leaves	GC-FID and GC-MS analysis	Estragole, *trans*-anethole, β-caryophyllene, myrcene	NI	FZL	ND	20 ± 0.5 8 ± 0.5 20 ± 0.5 8 ± 0.3	[42]

^a^ C. Conc = compound concentration. ^b^ Antifungals: FZL = fluconazole; MCZL = miconazole; VOZL = voriconazole; CFGN = caspofungin, AmphoB = amphotericin B. ^c^ MIC = minimum inhibitory concentration. ^d^ NI = not informed.

## Abbreviations

NI	not informed in the article
MIC	minimum inhibitory concentration
MFC	minimum fungicidal concentration
MW	molecular weight
HBD	hydrogen-bond donor
HBA	hydrogen-bond acceptor
LogP	values of lipophilicity
MF	molar refractivity
LD 50	lethal dose 50
TNF	tumor necrosis factor
IL	interleukin
